# Origin Matters: Differences in Embryonic Tissue Origin and Wnt Signaling Determine the Osteogenic Potential and Healing Capacity of Frontal and Parietal Calvarial Bones

**DOI:** 10.1359/jbmr.091116

**Published:** 2009-11-23

**Authors:** Natalina Quarto, Derrick C Wan, Matt D Kwan, Nicholas J Panetta, Shuli Li, Michael T Longaker

**Affiliations:** 1 Department of Surgery, Hagey Laboratory for Pediatric Regenerative Medicine, School of MedicineStanford, CA, USA; 2 Institute for Stem Cell Biology and Regenerative Medicine, School of MedicineStanford, CA, USA; 3 Department of Structural and Functional Biology, University of Naples Federico II Complesso M. S. AngeloNapoli, Italy

**Keywords:** tissue, origin, osteogenesis, Wnt signaling

## Abstract

Calvarial bones arise from two embryonic tissues, namely, the neural crest and the mesoderm. In this study we have addressed the important question of whether disparate embryonic tissue origins impart variable osteogenic potential and regenerative capacity to calvarial bones, as well as what the underlying molecular mechanism(s). Thus, by performing in vitro and in vivo studies, we have investigated whether differences exist between neural crest–derived frontal and paraxial mesodermal–derived parietal bone. Of interest, our data indicate that calvarial bone osteoblasts of neural crest origin have superior potential for osteogenic differentiation. Furthermore, neural crest–derived frontal bone displays a superior capacity to undergo osseous healing compared with calvarial bone of paraxial mesoderm origin. Our study identified both in vitro and in vivo enhanced endogenous canonical Wnt signaling in frontal bone compared with parietal bone. In addition, we demonstrate that constitutive activation of canonical Wnt signaling in paraxial mesodermal–derived parietal osteoblasts mimics the osteogenic potential of frontal osteoblasts, whereas knockdown of canonical Wnt signaling dramatically impairs the greater osteogenic potential of neural crest–derived frontal osteoblasts. Moreover, fibroblast growth factor 2 (FGF-2) treatment induces phosphorylation of GSK-3β and increases the nuclear levels of β-catenin in osteoblasts, suggesting that enhanced activation of Wnt signaling might be mediated by FGF. Taken together, our data provide compelling evidence that indeed embryonic tissue origin makes a difference and that active canonical Wnt signaling plays a major role in contributing to the superior intrinsic osteogenic potential and tissue regeneration observed in neural crest–derived frontal bone. © 2010 American Society for Bone and Mineral Research.

## Introduction

The vertebrate dermal skull roof is an ancient structure formed from membranous bones that are evolutionarily derived from the protective dermal plates of early jawless fishes.(1) Bones of the cranial vault form through the process of intramembranous ossification. Calvarial bones arise from two embryonic tissues, namely, the neural crest and the mesoderm. The distinct contributions of each tissue to the skull have been well established by combining mice with a *Wnt1-Cre* construct and a conditional reporter gene, *R26R.*(2,3) These studies have defined the pattern of cranial neural crest cell migration in mouse embryos and demonstrated that the frontal bone is of neural crest origin, whereas the parietal bone is of mesoderm origin. The neural crest (NC) is a population of cells unique to the vertebrate embryo.(4–6) NC cell (NCC) progenitors originate from the neural plate border and migrate into the periphery to contribute to multiple lineages.(7–9) In all vertebrates, a large part of the skull and the entire facial skeleton are derived from cephalic NCCs.

During development of the skeleton and formation of bone tissue, several morphogenetic growth factor and hormone signaling pathways impinge on transcriptional regulators to induce the osteogenic phenotype.(10,11) The challenge is to identify how developmental cues and regulatory factors are integrated to accommodate the requirements for biologic control of cell differentiation and tissue formation.

The Wnt signaling pathway is an important regulator of cellular differentiation in a variety of cell types, including osteoblasts.(12–16) It plays a widespread role in skeletogenesis, spanning from embryonic skeletal patterning through fetal skeletal development and bone remodeling.(17,18) Wnt proteins act on target cells by binding to Frizzleds (Fzs), seven-span transmembrane receptor proteins, and LRP-5/6, single-span transmembrane coreceptor proteins. The Wnt/β-catenin pathway is commonly referred to as the *canonical Wnt pathway* and is the most extensively studied pathway. The canonical Wnt pathway is initiated by the binding of appropriate Wnt ligands to the Fzs and LRP-5/6 coreceptor.(14,19,20) In the absence of appropriate ligands, β-catenin is phosphorylated at its NH_2_-terminal degradation box, leading to subsequent polyubiquitination and degradation.(14) However, on binding of an appropriate Wnt ligand to the receptor complex, the intracellular protein Dishevelled (Dvl) is activated.(21) Activation of Dvl leads to the inhibition of glycogen synthase kinase 3β (GSK-3β (22–24)), preventing β-catenin degradation by the protein complexes consisting of GSK-3β, axin, and adenomatous polyposis coli (APC).(23) Hence, since β-catenin cannot be targeted for degradation, cytoplasm accumulation transpires, followed by subsequent translocation to the nucleus, where in concert with members of the T cell factor/lymphoid enhancer factor (TCF/LEF) family, it activates transcription of a wide range of genes, including c*-myc*, *cyclin D1*, and *axin-2*.(25–27)

Studies on embryonic skeletogenesis have demonstrated that Wnt/β-catenin signaling represents a mechanism in mesenchymal precursor cells for selecting between chondrocytic and osteoblastic fates. Experiments involving both loss- and gain-of-function β-catenin alleles demonstrated that β-catenin is required to repress chondrogenesis and that in the absence of β-catenin, progenitor cells differentiated into chondrocytes rather than of osteoblasts.(28,29) In addition, Wnt/β-catenin signaling regulates bone density by activating osteoblasts and inhibiting osteoclasts.(30–32) Moreover, mutations in Wnt genes and receptors and inhibitors of the Wnt signaling pathway can have deleterious effects on normal bone formation and turnover, resulting in skeletal abnormalities. An activating mutation in the Wnt coreceptor *LRP5* results in the high-bone-mass trait in humans, a phenotype that is reproduced in the mouse model.(33–35) Mutations in *LRP6* also have been associated with a low-bone-formation phenoptype.(36) Loss-of-function mutations in LRP5 result in a low bone mass associated with decreased de novo bone formation.(30,37,38) Furthermore, *Wnt3a* knockout mice display a severe skeletal phenotype.(39–41)

Despite possessing a significant amount of knowledge regarding differences in skeletal regenerative potential between young and old mammals, we have essentially no information regarding the parity or dissimilarity of the osteogenic potential and endogenous skeletal regenerative capacity between calvarial bone arising from tissues of different embryonic origin. Establishing differences at both the cellular and molecular levels will be critical to the development of novel therapeutic approaches to enhance bone regeneration. In this study we have addressed the important question of whether disparate embryonic tissue origins impart variable osteogenic potential and regenerative capacity on calvarial bones, as well as what is the underlying molecular mechanism(s) might be. By performing in vitro and in vivo studies, we have demonstrated that calvarial bone osteoblasts of neural crest origin have superior potential for osteogenic differentiation. Furthermore, our data indicate that the neural crest–derived frontal bone displays a superior capacity to undergo osseous healing compared with calvarial bone of paraxial mesoderm origin. To this end, several lines of evidence indicate that these observations may be attributable at least in part to enhanced activation of canonical Wnt signaling present in the neural crest–derived frontal bone.

## Materials and Methods

### Animals/creation of calvarial defects/evaluation of calvarial healing

All experiments using animals were performed in accordance with Stanford University Animal Care and Use Committee guidelines. CD-1 wild-type mice were purchased from Charles River Laboratories, Inc. (Wilmington, MA, USA). Creation of calvarial defects and evaluation of calvarial healing in frontal and parietal bones are described in details under “Supplemental Materials and Methods.” frontal and parietal primary osteoblast culture and osteogenic differentiation Calvarial osteoblasts were harvested from the frontal and parietal bones of postnatal p7 (pN7) and pN60 mice. Isolation of osteoblasts and osteogenic differentiation conditions can be found under “Supplemental Materials and Methods.”

### Alkaline phosphatase activity and mineralization assay

Alkaline phosphatase enzymatic activity and alizarin red and von Kossa staining were performed as described previously.(42) All values were normalized against protein concentration obtained from triplicate wells.

### Transfections and infections

Details about infection and transfection of frontal and parietal osteoblasts with Dn-Tcf-4 and S33Y β-catenin(42,43) are described under “Supplemental Materials and Methods.”

### RNA isolation and reverse-transcriptase polymerase chain reaction (RT-PCR) analysis

Procedures for tissue harvesting, RNA isolation, and reverse transcription were described previously.(42) PCR reactions were performed under the following conditions: 94°C for 5 minutes, 94°C for 30 seconds, and annealing at 60°C for 1 minute and 72°C for 1 minute (25 to 30 cycles). Specific primers for the genes examined were designed based on their GenBank sequences. Primers sequence are as follows: mAxin F: CTCCCCACCTTGAATGAAGA; mAxin R: ACTGGGTCGCTTCTCTTGAA; mCyclin D1 F: TGGAGCCCCTGAAGAAGAG; mCyclinD1 R: AAGTGCGTTGTGCGGTAGC; and mMyc F: CTGTTTGAAGGCTGGATTT; mMyc R: TCGAGGTCATAGTTCCTGTT. Primer sequence and PCR conditions for the *Gapdh*, *Runx2*, *Alp*, and *osteocalcin* genes have been described previously.(44) For the densitometry analysis of RT-PCR, bands were scanned and quantified by using the ImageJ Program 1.36b (NIH, Bethesda, MD, USA). The densitometry results of each band were normalized to their respective loading control (*Gapdh*) and presented as percentage increase. The results are presented as the mean ± SD of the three independent experiments. Quantitative real-time RT-PCR was performed as described previously.(45) Primer sequences for *axin*, *myc*, and *cyclin D1* were described earlier; primer sequences and conditions for *Runx2*, *Alp*, and *osteocalcin* have been described previously.(46) The results are presented as means ± SD of two or three independent experiments.

### Statistical analysis

The results are presented as the mean ± SD of two or three independent experiments performed on different littermates. Statistical differences between the means were examined by Student's *t* test, and significance was set at a *p* value of less than .05.

### Western blot analysis

Procedures for β-catenin and GSK-3β immunodetection are provided under “Supplemental Materials and Methods.” Nuclear fractions were prepared as described previously.(47) Each nuclear fraction was analyzed by Western blotting using mouse anti-β-catenin antibody. To control for equal loading and transfer of the samples, the membrane also was reprobed with mouse monoclonal anti-lamin B1 nuclear envelop marker antibody (119D5-F1), dilution 1:200 (abcam.com, Cambridge, MA, USA). The densitometric results of each band were normalized to their respective loading controls (β-tubulin and lamin B1) and presented as a percentage increase.

### FGF-2 stimulation of parietal osteoblasts

Subconfluent osteoblasts were stimulated with 20 ng/mL of recombinant human fibroblast growth factor 2 (FGF-2; sc-4573, Santa Cruz Biotechnology, Santa Cruz, CA, USA) for 6, 12, and 24 hours. Before stimulation, cells were serum-starved for 12 hours in α-minimal essential medium (α-MEM) supplemented with 1% fetal calf serum (FCS). At each time point, cells were collected and nuclei were purified as described previously.(47) Then 80 µg of each nuclear fraction was analyzed by Western blotting technique using anti-β-catenin antibody as described earlier.

### Immunofluorescent staining, X-gal staining, and pentachrome staining

Procedures for indirect immunofluorescence staining, and histochemistry are provided under “Supplemental Materials and Methods.”

## Results

### Frontal and parietal osteoblasts have different osteogenic potential in vitro

**Fig. 1:**
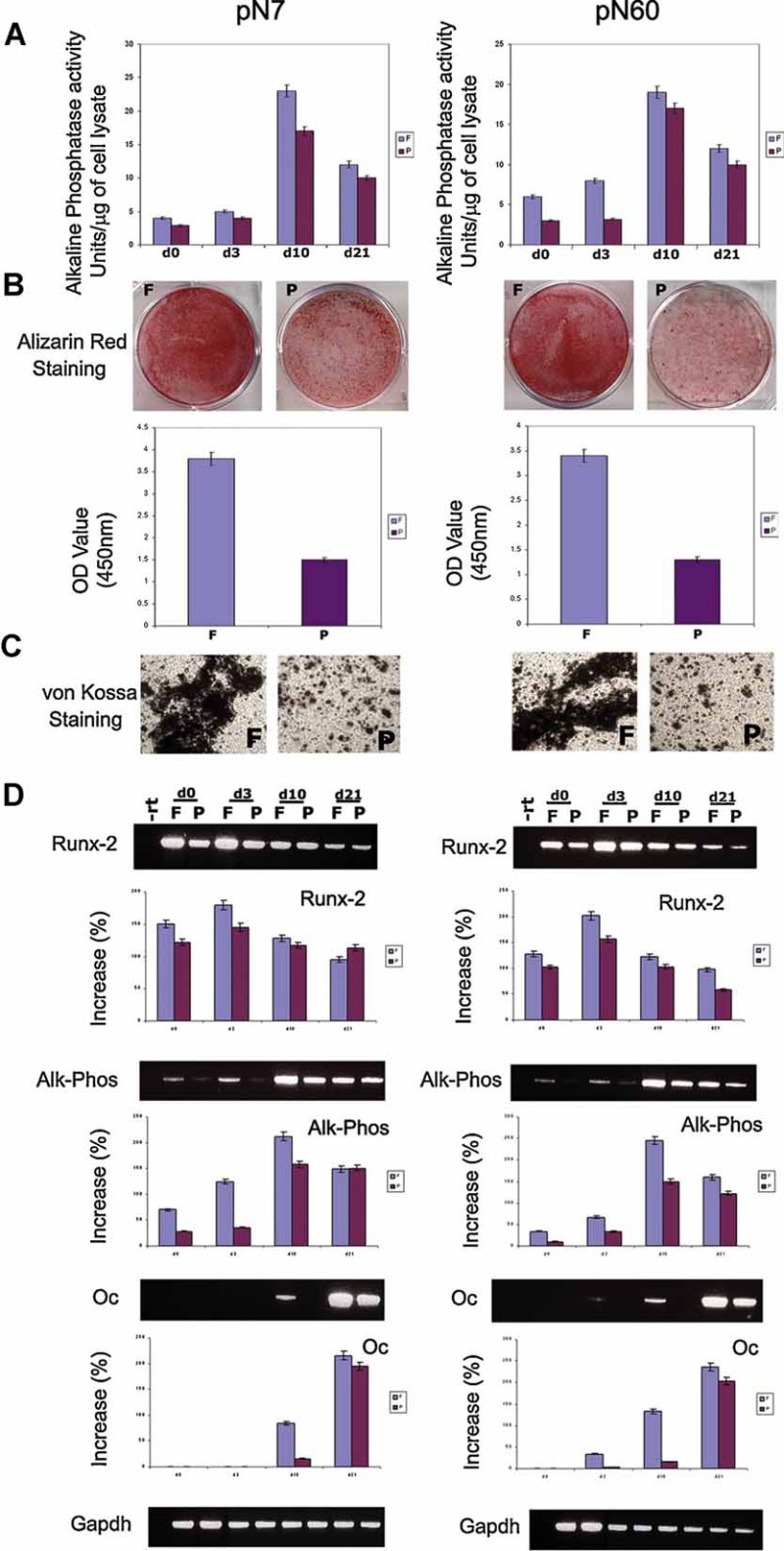
In vitro osteogenic potential of neural crest–derived frontal bone and paraxial mesoderm–derived parietal bone osteoblasts. (*A*) Alkaline phosphatase activity in pN7 and pN60 frontal and parietal bone osteoblasts. Elevated levels of enzymatic activity were observed in frontal bone osteoblasts. (*B*) Alizarin red staining and its quantification detected more intense mineralization of extracellular matrix in frontal bone osteoblasts (20× objective magnification). Abbreviations: *F* = frontal; *P* = parietal. (*C*) von Kossa staining revealed the presence of bone nodules of larger size in frontal bone osteoblasts. (*D*) RT-PCR analysis of osteogenic markers *Runx-2, Alp* (Alk Phos), and *osteocalcin* (Oc) in frontal and parietal bone osteoblasts. –rt is a negative control for the reverse transcription reaction performed on pooled RNAs purified from each experimental point. Histogram represents the densitometric analysis of RT-PCR products performed using ImageJ software; the relative intensities of bands were normalized to their respective loading control (*Gapdh*) and set as 100%; The results are presented as the mean ± SD of three independent experiments.

To assess the osteogenic potential of frontal bone– and parietal bone–derived osteoblasts, we established primary cell culture of osteoblasts harvested from the two different bones isolated in postnatal days 7 (pN7) and 60 (pN60) mice. In order to ensure the accuracy of dura mater and pericranium removal from frontal and parietal bones prior to their enzymatic digestion, we optimized the dissecting procedure using *Wnt1-Cre/R26R* mice. This is a transgenic mouse with a permanent neural crest cell lineage marker that allows X-gal staining of neural crest–derived tissues (ie, pericranium, frontal bone, and dura mater) (Supplemental Fig. 1). Osteogenic differentiation was investigated on first-passage osteoblasts isolated from frontal and parietal bones. Alkaline phosphates activity showed that pN7 and pN60 frontal bone–derived osteoblasts displayed higher enzymatic activity (Fig. 1*A*). Long-term differentiation of osteoblasts in vitro induces matrix production, followed by mineralization of the extracellular matrix. To assess for mineralization of extracellular matrix and bone nodules formation, alizarin red and von Kossa staining was performed on days 21 and 28 of osteogenic differentiation. As shown in Fig. 1*B, C*, more intense mineralization of extracellular matrix, as well as larger bone nodules formation, was observed in frontal than in parietal osteoblast cultures. To further examine the osteogenic potential of frontal and parietal osteoblasts, the temporal expression of early, intermediate, and late osteoblast-specific markers *Runx2*, *Alp*, and *osteocalcin* was determined by RT-PCR analysis (Fig. 1*D* and Supplemental Fig. 2). The analysis revealed that in frontal osteoblasts the expression of the late marker *osteocalcin* was prematurely elevated, and the expression of *Alp* also was increased, in agreement with the enzymatic activity. Interestingly, as observed previously in freshly harvested frontal and parietal tissues,(48) frontal bone–derived osteoblasts also elaborated higher levels of *Runx2* (Fig. 1*D*). The latter observation would suggest the presence of a larger pool of osteoprogenitor cells in the frontal bone. Taken together the data reveal distinct differences in in vitro osteogenic potential between frontal bone– and parietal bone–derived osteoblasts.

**Fig. 2:**
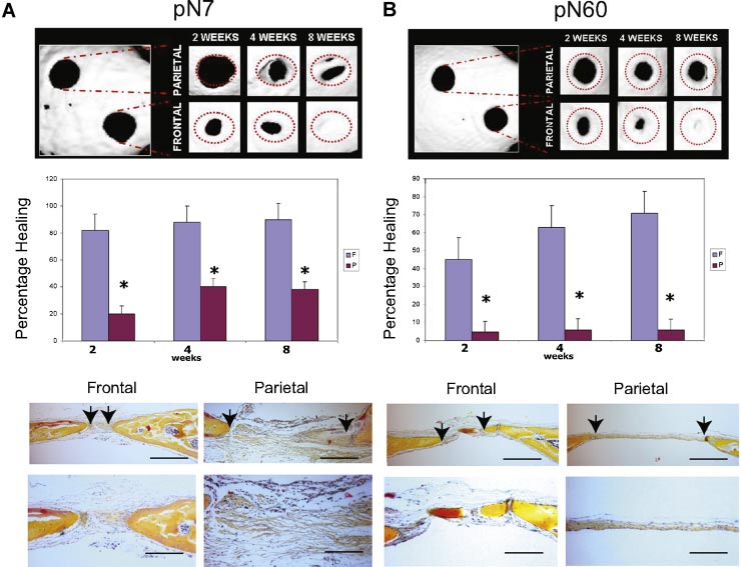
In vivo osteogenic potential of frontal and parietal bones. (*A*) Two-millimeter calvarial defects were made in the frontal and parietal bones of pN7 mice (*upper panel*). µCT performed at 2, 4, and 8 weeks after osteotomy. Quantification of bone regeneration (*middle panel*) (*p ≤ .05). Pentachrome staining (*lower panel*). (*B*) Two-millimeter calvarial defects were made in the frontal and parietal bones of pN60 mice (*upper panel*). Quantification of bone regeneration (*middle panel*) (*p ≤ .05). Pentachrome staining (*lower panel*). Arrows indicate the edges of defect. Scale bars = 150 µm in 20× magnification panels, 50 µm in 40× magnification panels.

### In vivo potential for healing of calvarial defects in mouse neural crest–derived frontal and paraxial mesoderm–derived parietal calvarial bones

The preceding observation prompted us to investigate whether regional differences in in vivo osteogenic potential exist by asking two specific questions: (1) Are there differences in the rate of calvarial healing between neural crest–derived frontal bones and paraxial mesoderm–derived parietal bones of juvenile (pN7) mice? and (2) If differences exist, do they persist in adult mice (pN60)? Therefore, 2-mm calvarial defects were created in the frontal and parietal bones of pN7 juvenile mice to determine whether differences in the rate of healing exist between the frontal and parietal bones (Fig. 2*A*). Subsequently, 2-mm calvarial defects were created in the frontal and parietal bones of pN60 adult mice (Fig. 2*B*) to establish whether any differences in the rate of healing observed in juvenile mice persist in mature calvarial skeletal tissue. Micro–computed tomography (µCT) revealed increased healing of neural crest–derived frontal bone defects compared with parietal bone defects in pN7 and pN60 mice throughout the course of the investigation (Fig. 2*A, B*). Histologic analysis performed on coronal sections using pentachrome staining revealed the presence of osseous regenerate bridging at the injury site with new bone formation centrally in the frontal bone defects after 8 weeks, consistent with successful bone healing. In contrast, significant healing of the parietal bone was not detected, and a wide gap between the osteogenic fronts of the two bone plates surrounding the area of injury was noticed. These results corroborate our in vitro observations, putting forth further evidence that indeed differences in embryonic calvarial bone origin impart differences in the potential for healing of subsequently created to defects in juvenile as well as adult mice.

### Enhanced activation of canonical Wnt signaling in frontal bone-derived osteoblasts

**Fig. 3:**
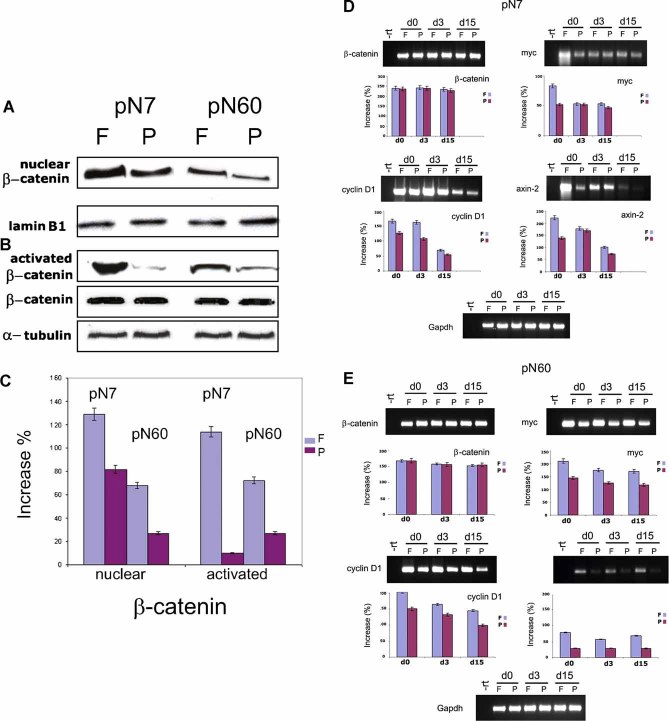
Enhanced activation of canonical Wnt signaling in frontal bone–derived osteoblasts and frontal bone. (*A*) Western blotting analysis revealed higher levels of nuclear β-catenin in both pN7 and pN60 frontal bone osteoblasts than in parietal bone osteoblasts. Membranes were stripped and reprobed with anti-Lamin B1 antibody to assess for equal loading and transfer of nuclear protein fractions. (*B*) Pool of active nonphosphorylated β-catenin was assessed on whole-cell lysates using anti-β-catenin antibody, which specifically detects nonphosphorylated active β-catenin. The membranes were stripped and subsequantially incubated with pan-β-catenin antibody and α-tubulin antibody to assess for the total amount of endogenous β-catenin and to control for equal loading and transfer of the samples. (*C*) Histogram represents the densitometric analysis of electrophoresis bands; the relative intensities of bands were normalized to their respective loading control and set as 100%. The results are presented as the mean ± SD of three independent experiments. (*D*) Expression level of endogenous *β*-*catenin*, *cyclin D1*, *myc*, and *axin-2* in pN7 frontal and parietal bone osteoblasts. (*E*) RT-PCR analysis of endogenous *β-catenin*, *cyclin D1*, *myc*, and *axin-2* in pN60 frontal and parietal osteoblasts. Histogram represents the densitometric analysis of RT-PCR products performed as above. The results are presented as the mean ± SE of three independent experiments.

Our observation that differences in embryonic tissue origin between calvarial osteoblasts of neural crest and paraxial mesoderm translate into regional differences in osteogenic potential and calvarial healing capacity engendered interest to investigate the molecular mechanism(s) underlying this phenomenon. Several prior investigations supported a widespread role of canonical Wnt signaling in skeletogenesis and bone remodeling.(17,18) Therefore, we set out to explore the relationship between observed differences in frontal bone– and parietal bone–derived osteoblast biology and Wnt signaling. To begin, endogenous Wnt signaling was measured in pN7 and pN60 frontal and parietal bone osteoblasts. To directly assess the signaling pool of β-catenin, we investigated nuclear levels of β-catenin as well as levels of active β-catenin. Western blot analyses showed increased levels of nuclear β-catenin (Fig. 3*A, C*), as well as higher levels of active β-catenin in frontal bone osteoblasts compared with parietal bone osteoblasts (Fig. 3*B, C*). In contrast, no significant differences in endogenous levels of total β-catenin were observed (Fig. 3*B*).

It is well established that a functional consequence of β-catenin nuclear translocation is its association with Lef/Tcf proteins and resulting induced transcription of target genes, including *myc*, *cyclin D1*, and *axin-2*.(25–27) To further investigate canonical Wnt signaling activation, we analyzed the expression levels of *myc*, *cyclin D1*, and *axin-2* in frontal and parietal bone osteoblasts. As shown in Fig. 3*D, E*, higher levels of *myc*, *cyclin D1*, and *axin-2* were observed in pN7 and pN60 frontal bone–derived osteoblasts on day 0 of osteogenic differentiation. During osteogenic differentiation, the expression of all three target genes downregulated in frontal osteoblasts. In contrast, only *cyclin D1* decreased over time in parietal bone–derived osteoblasts, whereas *myc* expression remained steady. *Axin-2* slightly upregulated at an intermediate point of osteogenic differentiation in pN7 osteoblasts, followed by a decrease in expression on day 15. These expression patterns also were confirmed by quantitative RT-PCR analysis (Supplemental Fig. 3) Collectively, these data show that in frontal bone osteoblasts there is an enhanced endogenous activation of canonical Wnt signaling relative to parietal osteoblasts.

**Fig. 4:**
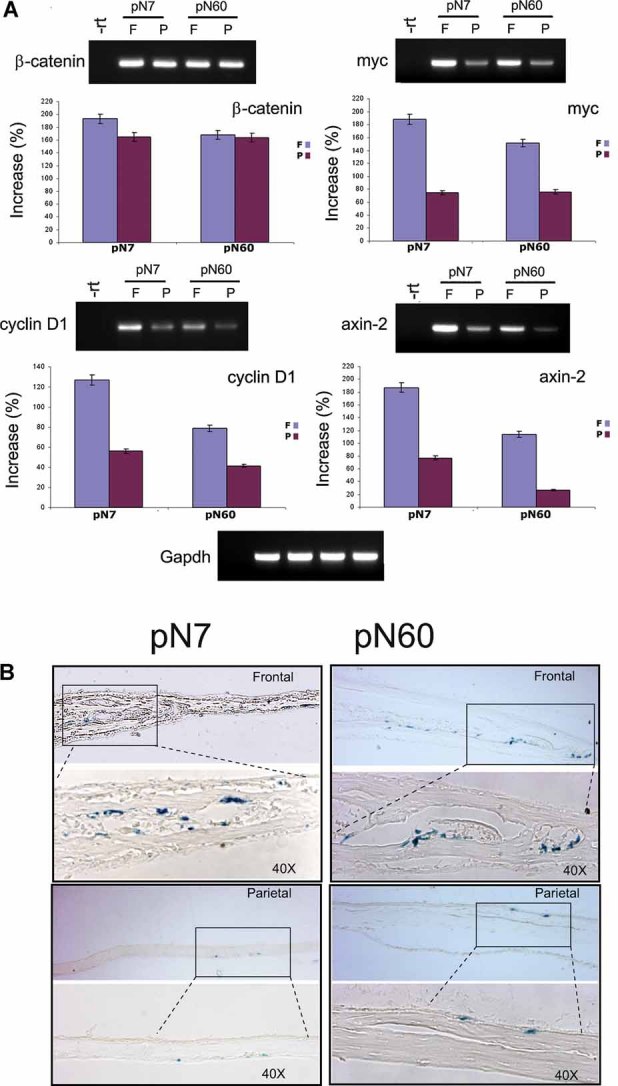
Enhanced activation of canonical Wnt signaling in neural crest–derived frontal bone. (*A*) RT-PCR analysis of endogenous *β-catenin*, *cyclin D1*, *myc*, and *axin-2* in frontal and parietal bones. Histogram represents the densitometric analysis of RT-PCR products performed as above. (*B*) X-gal staining of coronal sections of frontal and parietal bones of *axin-2^lacZ/+^* reporter mice showed more intense staining of frontal bone as result of an enhanced activation of canonical Wnt signaling. Boxed areas are enlarged in the lower panels (40×).

We next investigated whether differences in the activation of canonical Wnt signaling persist in vivo between frontal and parietal bones. To probe this possibility RT-PCR analysis of the downstream canonical Wnt signaling target genes was performed on frontal and parietal bone tissues. In agreement with the in vitro results, this analysis revealed higher levels of *myc*, *cyclin D1*, and *axin-2* in frontal bone, thus confirming the occurrence of enhanced activation of canonical Wnt signaling in the neural crest–derived frontal bone (Fig. 4*A* and Supplemental Fig. 4*A–D*). Because *axin-2* is transcriptionally induced following reception of Wnt/β-catenin signal,(27) to further monitor the activation of Wnt signaling in frontal and parietal bones, we used *axin-2*^*lacZ/+*^ mice in which a *lacZ* reporter gene has been inserted into the Wnt target gene *axin-2*.(49) Here, X-gal staining monitors activation of Wnt signaling. The X-gal staining revealed substantially higher levels of *axin-2* in the frontal bone compared with the parietal bone in both pN7 and pN60 mice; *axin-2* expression was identified in osteoblasts, periosteum, and dura mater (Fig. 4*B*). Thus in vivo data were in agreement with the in vitro observation.

### Constitutive activation of canonical Wnt signaling in parietal bone osteoblasts mimics the osteogenic potential of frontal bone osteoblasts

**Fig. 5:**
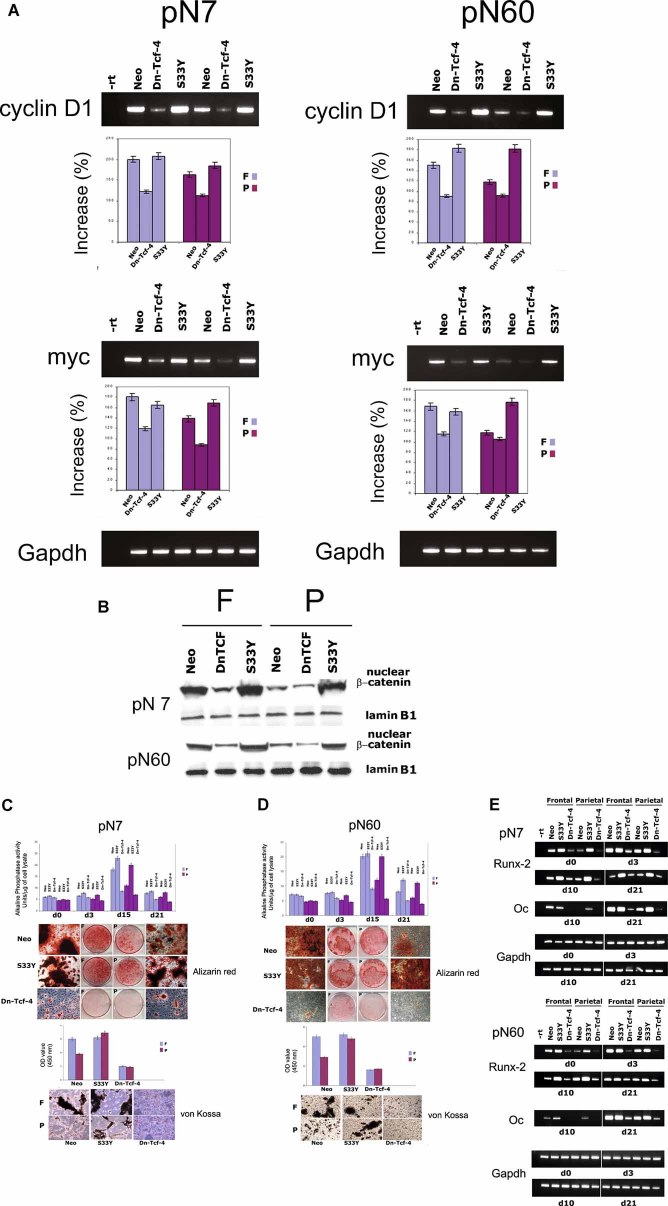
Constitutive activation of canonical Wnt signaling in parietal bone osteoblasts mimics the osteogenic potential of frontal bone osteoblasts. (*A*) RT-PCR analysis indicated that either in pN7 or pN60 S33Y-infected parietal bone osteoblasts the expression of *myc* and *cyclin D1* genes achieved threshold levels similar to those of control Neo frontal bone osteoblasts. In contrast, expression of Dn-Tcf-4 drastically downregulated the endogenous levels of *myc* and *cyclin D1* in frontal bone osteoblast and parietal bone osteoblasts. (*B*) Western blotting analysis revealed increased nuclear β-catenin in S33Y-infected parietal bone osteoblasts with levels similar to that of Neo control frontal bone osteoblasts. Decreased accumulation of nuclear β-catenin was observed in both Dn-Tcf-4-infected frontal and parietal bone osteoblasts compared with controls. Filters were stripped and reprobed with anti-Lamin B1 antibody. (*C*, *D*) Osteogenic profile determined by alkaline phosphatase activity and alizarin red and von Kossa staining. (*E*) RT-PCR analysis of osteogenic markers during osteogenic differentiation.

Taken together the data described to this point led us to hypothesize that enhanced activation of canonical Wnt signaling in frontal bone osteoblasts may confer greater osteogenic potential to these cells. In order to verify this hypothesis, we both activated and inhibited canonical Wnt signaling in frontal and parietal bone osteoblasts. To this end cells were infected either with the retrovirus pPGS expression dominant-negative Tcf-4 (Dn-Tcf-4) or S33Y, a β-catenin mutant that accumulates in the nucleus and constitutively activates TCF-mediated transcription-activated β-catenin.(43) An empty pPGSNeo vector was implemented as control. First, we confirmed that DnTcf-4 could inhibit TCF-mediated transcription, whereas constitutively active β-catenin could by itself activate TCF-mediated transcription. For this purpose, each type of infected cell was analyzed for the expression levels of downstream Wnt signaling target genes. RT-PCR analysis demonstrated that in S33Y parietal osteoblasts, the expression levels of downstream Wnt target genes *myc* and *cyclin D1* were high, approximating levels observed in control Neo frontal bone osteoblasts (Fig. 5*A*). In contrast, both frontal and parietal bone osteoblasts infected with the Dn-Tcf-4 retroviral plasmid expressed lower levels of target genes as result of the inhibition of Wnt signaling (Fig. 5*A*). These results also were confirmed by a quantitative RT-PCR analysis (Supplemental Fig. 4*E–H*). To further confirm the constitutive activation, as well inhibition, of canonical Wnt signaling in frontal and parietal bone retrovirally infected osteoblasts, we analyzed the amount of nuclear β-catenin by Western blot technique. As shown in Fig. 5*B*, in both pN7 and pN60 Dn-Tcf-4 frontal bone osteoblasts, the nuclear pool of β-catenin markedly decreased to levels similar to that of control parietal osteoblasts. Conversely, in S33Y parietal bone osteoblasts, the amount of nuclear β-catenin was similar to that of control frontal bone osteoblasts. Moreover, it was observed that the pool of nuclear β-catenin also increased in S33Y frontal bone osteoblasts relative to control frontal bone osteoblasts, and expression of Dn-Tcf-4 decreased the endogenous level of nuclear β-catenin in both frontal and parietal bone osteoblasts.

Thus, having established the occurrence of a constitutive activation of canonical Wnt signaling in S33Y-infected osteoblasts, as well as its inhibition in Dn-Tcf-4-infected osteoblasts, we next addressed whether enhanced activation of canonical Wnt signaling in parietal osteoblasts could mimic the greater osteogenic potential observed in frontal bone osteoblasts and vice versa, whether an inhibition of canonical Wnt signaling in frontal osteoblasts could make them more “parietal bone–like.” To this end, the retrovirally infected osteoblasts were tested for their osteogenic potential. As shown in Fig. 5*C, D*, S33Y-infected parietal osteoblasts displayed an osteogenic potential similar to that of control frontal bone osteoblasts, characterized by increased alkaline phosphatase activity and increased mineralization of extracellular matrix. In contrast, Dn-Tcf-4-infected frontal bone osteoblasts displayed an osteogenic pattern similar to that of control Neo parietal bone osteoblasts, characterized by decreased alkaline phosphatase activity and decreased mineralization of extracellular matrix. In addition, the expression level of early and late bone-related genes was examined by RT-PCR (Fig. 5*E*). In addition, higher levels of the osteogenic markers *Runx-2* and *osteocalcin* were observed in S33Y-infected parietal bone osteoblasts compared with Neo parietal bone osteoblasts. These results demonstrated that enhancing the activation of canonical Wnt signaling in parietal bone osteoblasts imparted a more robust osteogenic potential similar to that of neural crest–derived frontal bone osteoblasts. On the contrary, downregulation of canonical Wnt signaling in frontal bone osteoblasts impaired their superior osteogenic potential. Thus the data strongly support the hypothesis that enhanced canonical Wnt signaling may play a crucial role in the higher osteogenic potential of frontal bone–derived osteoblasts.

### Exogenous added Wnt3a confers a higher osteogenic potential to parietal bone osteoblasts

**Fig. 6:**
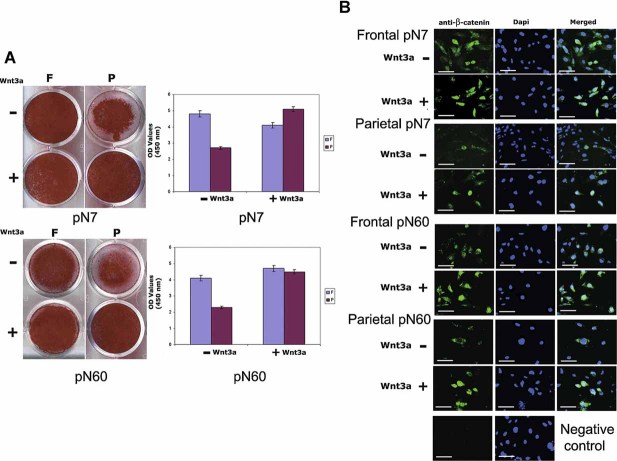
Exogenous added Wnt3a confers higher osteogenic potential to parietal bone osteoblasts. (*A*) Osteogenic differentiation of frontal and parietal bone pN7 and pN60 osteoblasts in the presence or absence of 50 ng/mL Wnt3a assessed by alizarin red staining. (*B*) Indirect immunofluorescence staining detected intense nuclear staining in Wnt3a-treated parietal bone osteoblasts compared with untreated parietal bone osteoblasts. Increased nuclear translocation of β-catenin also was observed in treated frontal bone osteoblasts, although in a less dramatic manner than in parietal bone osteoblasts. As negative control, a normal primary (irrelevant) mouse IgG was used. Nuclear counterstaining was performed with DAPI (objective magnification 10×). Scale bars = 50 µm.

One might speculate that constitutive activation of β-catenin achieved in S33Y-infected osteoblasts represents a condition that bypasses the initial step of activation of canonical Wnt signaling (eg, cell surface receptor-ligand interaction) and therefore does not provide a complete clue about the capability of parietal bone osteoblasts in transducing Wnt signaling. To investigate the ability of parietal bone osteoblasts to respond to Wnt stimuli and to further demonstrate that the activation of canonical Wnt signaling is responsible for the greater osteogenic capacity of frontal bone osteoblasts, the osteogenic potential of frontal and parietal bone osteoblasts was assayed in the presence of exogenous added Wnt3a. Wnt3a was chosen secondary to the abundance of data implicating this Wnt ligand in regulation of bone maintenance and osteogenesis both during development and in adult animals. Addition of Wnt3a at concentration of 50 ng/mL to osteogenic medium resulted in an increased differentiation on both frontal and parietal bone osteoblasts, with the latter showing mineralization of extracellular matrix comparable with that of untreated frontal bone osteoblasts (Fig. 6*A*). Moreover, immunofluorescence analysis revealed that exposure of parietal bone osteoblasts to exogenous Wnt3a mediated a strong activation of β-catenin signaling. As shown in Fig. 6*B*, after 6 hours Wnt3a treatment, a major nuclear translocation of β-catenin was observed in parietal bone osteoblasts compared with untreated parietal bone osteoblasts. Moreover, intense nuclear staining was observed in untreated frontal bone osteoblasts, and the nuclear staining was further enhanced in the presence of exogenous Wnt3a.

These results led to two conclusions: First, parietal bone osteoblasts are responsive to exogenous Wnt stimuli, such as Wnt3a ligand, and therefore are capable of transducing an external signal, and second, activation of canonical Wnt signaling on binding of Wnt3a ligand to the cell surface receptor(s) confers a higher osteogenic capacity to parietal bone osteoblasts that mimics that of frontal bone osteoblasts. These findings, paired with our data subsequent to retroviral infection, imply that active canonical Wnt signaling may play a major role in contributing to the superior intrinsic osteogenic potential observed in frontal bone osteoblasts.

### Differential activation of canonical Wnt signaling in frontal and parietal bones following injury

Recent published data revealed that the Wnt signaling pathway is activated during postnatal bone-regenerative events and that dysregulation of this pathway greatly inhibits bone-formation and healing processes.(50–52) Because our data indicated that the healing capacity of frontal bone is superior to that of parietal bone and also unveiled the presence an enhanced activated canonical Wnt signaling in frontal bone, we decided to investigate the extent to which of canonical Wnt signaling is activated in frontal versus parietal bone on injury. Thus 2-mm calvarial defects were drilled in the frontal and parietal bones of pN7 and pN60 *axin-2*^*lacZ/+*^ heterozygotic mice.(49) Intense X-gal staining was observed in injured frontal bones on postoperative day 5 (Supplemental Fig. 5). In contrast, the intensity of staining was substantially less in injured parietal bone staining (Supplemental Fig. 5). X-gal staining was detected in the frontal bone as early as 24 and 48 hours after injury, whereas staining was minimally present at the site of parietal bone at these time points (data not shown). These observations demonstrated that on injury, frontal bone responds with a more robust activation of canonical Wnt signaling than parietal bone.

### Frontal bone–derived osteoblasts are endowed with higher levels of inactivated GSK-3β than parietal bone–derived osteoblasts

**Fig. 7:**
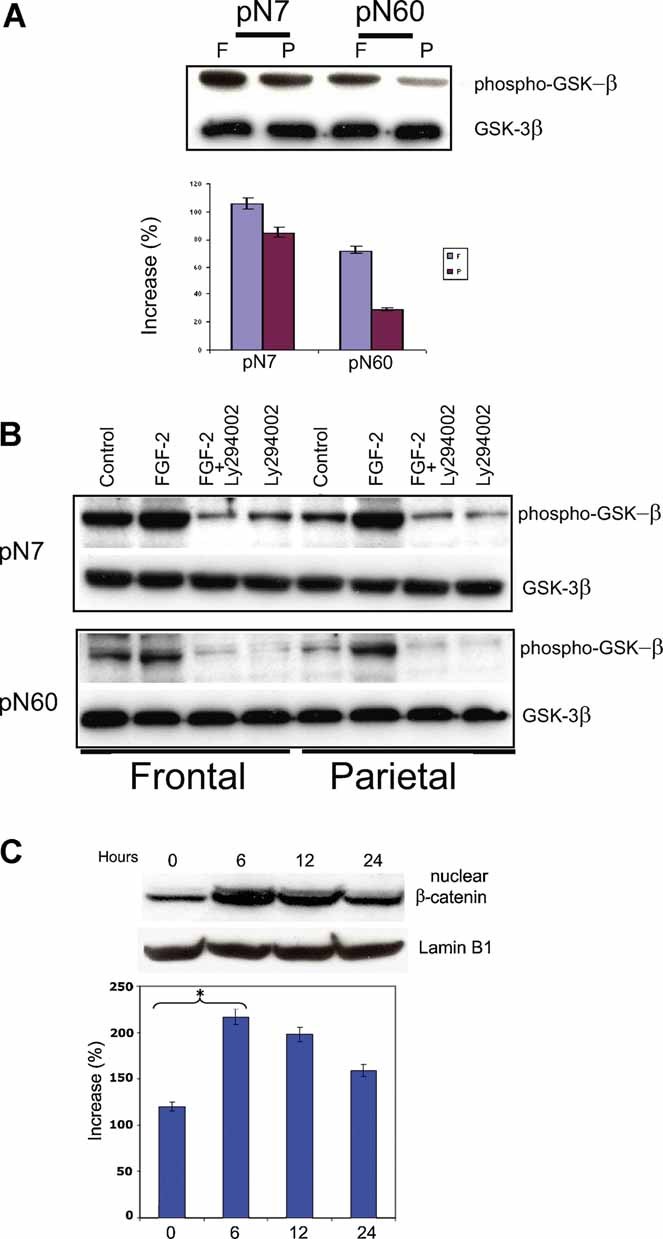
Frontal bone–derived osteoblasts are endowed with higher levels of inactivated GSK-3β than parietal bone–derived osteoblasts. (*A*) Western blot analysis of cell lysates using a polyclonal phospho-GSK-β3 antibody. Histogram represents the densitometric analysis of electrophoresis bands. (*B*) Western blot analysis of frontal and parietal bone osteoblasts treated with FGF-2 (20 ng/mL) indicated that FGF-2 treatment increases the pool of phosphorylated GSK-β3. FGF-2-induced phosphorylation of GSK-β3 is abrogated by the presence of Ly294002. Membranes were stripped and reprobed with a monoclonal anti-GSK-β3 antibody to ensure equal loading and transfer of proteins. (*C*) FGF-2 treatment enhances nuclear translocation of β-catenin. Immunoblotting analysis of nuclear fraction harvested from pN7 parietal bone osteoblasts stimulated with 20 ng/mL of FGF-2 at different time points. One of three independent experiments is shown. Densitometrical analysis revealed increase in nuclear β-catenin, with a peak at 6 hours of stimulation. The results are presented as the mean ± SD of three independent experiments (*p < .03). To show equal protein loading, membranes were stripped and reprobed with anti-Lamin B1 antibody.

The inactivation of GSK-3β leads to the nuclear accumulation of β-catenin.(53) Because our preceding data revealed an increased pool of nuclear/activated β-catenin in frontal bone–derived osteoblasts, we investigated whether variable amounts of active GSK-3β were present between frontal and parietal bone osteoblasts. Western blot analysis performed using an antiphospho-GSK-3β antibody that detects GSK-3β only when inactivated by phosphorylation at serine 9 revealed substantial differences in the endogenous levels of phosphorylated GSK-3β bewtween frontal and parietal bone osteoblasts, therefore confirming the inhibition of GSK-3β activity in the frontal bone (Fig. 7*A*).

Our previous study performed on both osteoblasts and tissues derived from frontal and parietal bones has highlighted an upregulation of FGF ligands such *Fgf-2, −9*, and *−18* and *Fgf R-1*, *−2*, and *−3* transcripts in the frontal bone of E17.5 pN1 and pN60 mice compared with parietal bone.(48) It is noteworthy that growth factors such as insulin, insulin-like growth factor 1 (IGF-1), and fibroblast growth factor 2 (FGF-2) inhibit GSK-3β activity by inducing its phosphorylation.(54–57) Because frontal bone displays higher threshold levels of FGF ligands and phosphorylated GSK-3β protein relative to parietal bone, we finally tested whether FGF-2 treatment could induce GSK-3β phosphorylation in parietal bone–derived osteoblasts leading to threshold levels of phosphorylated GSK-3β similar to that observed in frontal bone–derived osteoblasts. To this end, frontal and parietal bone osteoblasts were incubated for 3 hours with 20 ng/mL of FGF-2 and without FGF-2. Western blot analysis revealed that FGF-2 treatment increased the level of phosphorylated GSK-3β in parietal bone osteoblasts to that of untreated frontal bone osteoblasts (Fig. 7*B*). In addition, FGF-2 treatment further increased the level of phosphorylated GSK-3β in frontal bone osteoblasts. Previous findings indicated that phosphorylation GSK-3β by cytokines is mediated through the activation of the PI 3-K/Akt signaling pathway.(57) To verify whether FGF-2 induces phosphorylation of GSK-3β in osteoblasts through the PI 3-K/Akt pathway, cells also were incubated with Ly294002, a chemical-specific inhibitor of the PI 3-K/Akt pathway. As shown in Fig. 7*B*, in the presence of 50 µM Ly294002, FGF-2 did not phosphorylate GSK-3β; moreover, treatment with inhibitor alone decreased the amount of endogenous phosphorylated GSK-3β.

Next, we investigated whether FGF-2 treatment could induce nuclear accumulation of β-catenin. A time-course experiment performed on pN7 parietal bone osteoblasts reveled that stimulation with FGF-2 increased the level of nuclear β-catenin, with a peak at 6 hours of stimulation (Fig. 7*C*).

## Discussion

Knowledge regarding of the embryonic origins of mammalian frontal and parietal bones has prompted us to ask the question, “Do differences in embryonic tissue origin translate into differences in biologic activity on a cellular and molecular level?” To address this question, we have investigated, in vitro and in vivo, the osteogenic differentiation potential and calvarial healing capacity of both frontal and parietal bones. Remarkably, our data revealed that calvarial bone of neural crest origin has superior potential for osteogenic differentiation in vitro and osseous healing in vivo when compared with bone of paraxial mesoderm origin. These observations provide compelling evidence that indeed differences in embryonic tissue origin translate into variable potential for osteogenic differentiation as well as tissue regeneration. Moreover, we present evidence indicating that canonical Wnt signaling is enhanced in neural crest–derived frontal bone compared with parietal bone and that this signaling may determine the superior osteogenic potential of frontal bone versus parietal bone.

The first evidence supporting the enhanced activation of canonical Wnt signaling in frontal bone was the observation that the levels of nuclear and actived β-catenin were markedly increased in frontal bone osteoblasts of pN7 and pN60 mice. Additionally, RT-PCR analysis performed on osteoblasts, as well as on fresh tissues isolated from frontal and parietal bones, revealed higher expression of *myc*, *cyclin D1*, and *axin-2* in frontal bone, suggesting indeed enhanced activation of Wnt signaling in this bone.

A study by Guar and colleagues(58) has shown that in bone of *SFRP1* (secreted Frizzled-related protein1) null mouse, which exhibits enhanced activated Wnt signaling and a high-bone-mass phenotype, there was a significant increase in expression of *Runx-2* and the RUNX-2 target gene *osteocalcin*. Moreover, the authors demonstrated by a mutational analysis that a functional TCF regulatory element responsive to canonical Wnt signaling resides in the promoter of the *Runx-2* gene, thus suggesting that *Runx-2* is a direct target gene of the canonical Wnt pathway.(58) Notably, our data revealed upregulation of both endogenous *Runx-2* and canonical Wnt signaling in frontal bones, congruent with the superior osteogenic differentiation and bone tissue regeneration observed there. These results led us to propose that direct regulation of *Runx-2* gene expression in vivo by canonical Wnt signaling is a contributing factor to the greater osteogenic potential and skeletal regeneration of frontal bone in both juvenile and adult mice. To elucidate the involvement of canonical Wnt signaling in the greater osteogenic potential of frontal bone, we proceeded to both impair and augment the level of canonical Wnt signaling in both frontal or parietal bone osteoblasts. These experiments demonstrated that activation of canonical Wnt signaling in parietal bone osteoblasts could mimic the robust osteogenic potential of unperturbed frontal bone osteoblasts. Alternatively, inhibition of canonical Wnt signaling in frontal bone osteoblasts resulted in a dramatic decrease of their osteogenic potential.

Recent published data revealed the importance of the Wnt signaling pathway in bone-regenerative processes and activation during postnatal bone regeneration.(59–62) Moreover, Leucht and colleagues have reported that embryonic origin and *Hox* gene expression status distinguish neural crest–derived from mesoderm-derived skeletal progenitor cells, and both characteristics influence the process of adult bone regeneration.(63) Therefore, we explored the relationship between bone injury and activation of canonical Wnt signaling in frontal and parietal bones. Calvarial defects created on Axin-2^LacZ/+^ mice clearly indicated that on injury, a stronger activation of canonical Wnt signaling transpires in frontal bone compared with parietal bone. We believe that this enhanced activation of canonical Wnt signaling may contribute significantly to successful healing of frontal bone.

In the context of our study, it is noteworthy that null mutation of canonical Wnt pathway downstream target gene c*-myc* gave rise to a dramatic phenotype affecting the frontal bone. Wei and colleagues demonstrated that *Wnt1-Cre* inactivation of the c*-myc* gene resulted in a viable mouse displaying absence of frontal bone ossification.(64) This study strongly supports our hypothesis that canonical Wnt signaling significantly contributes to the observed superior osteogenic properties of neural crest–derived frontal bone.

Our previous study focused on comparative gene expression profiles of FGF ligands and their receptors in neural crest–derived frontal and paraxial mesoderm–derived parietal bones revealed a differential expression pattern of the major FGF osteogenic ligands and their receptors.(48) Furthermore, an independent ongoing study has revealed striking differences in the activation of the three FGF-mediated intracellular signaling pathways between neural crest–derived and paraxial mesoderm–derived osteoblasts, with a constitutive marked increase in the phosphorylation of Erk, PI3K/Akt, and PKC α, β, and γ in the frontal bone–derived osteoblasts (manuscript in preparation). These results suggest that the neural crest–derived frontal bone is also a domain of highly activated FGF signaling compared with parietal bone.

It must be pointed out that during our study, Western blot analysis did not detect differences at the protein level for at least two of the major Wnt ligands known to activate canonical Wnt signaling (Wnt3a and Wnt10; data not shown). Similarly, no significant differences were observed at the gene level for receptors, coreceptors, and inhibitors (data not shown). Therefore, the question of what activates β-catenin in frontal bone remains to be determined.

Several studies have indicated that growth factors, including insulin, IGF-1, FGF-2, and bone morphogenetic protein (BMP), stimulate the β-catenin-mediated Wnt signaling pathway through signaling involving GSK-3β inhibition.(54,55,65) Our data would suggest that the enhanced activation of canonical Wnt/β-catenin could be mediated by FGF ligands expressed at high levels in the frontal bone. In support of this hypothesis is the observed induction of phosphorylation of GSK-3β and augmentation of nuclear levels of β-catenin in parietal osteoblasts when treated with FGF-2, as well as the endogenous higher level of phosphorylated GSK-3β in frontal bone osteoblasts. However, our hypothesis deserves further investigation to rule out distinct molecular mechanism(s).

For instance, a study by McManus and colleagues(66) demonstrated that in double *GSK* knock-in mice in which the Ser9 in *GSK3β* and Ser21 in *GSKα* are mutated to alanine, Wnt-mediated inactivation of *GSK3* does not depend on phosphorylation of *GSK3α* at Ser21 and *GSKβ* at Ser9. Moreover, these knock-in mice develop normally. In this context, it would be interesting to analyze whether in pathologic conditions such as calvarial injury the *GSK* knock-in mice have impaired bone healing, as well as to test the effect of FGF-2 on inactivation of *GSK*.

In the light of our results from current and prior studies, we propose that the high FGF signaling domain present in frontal bone is a permissive condition for the enhanced activation of canonical Wnt signaling in neural crest–derived bone.

Several studies have indicated that substantial interplay and crosstalk between the FGF and canonical Wnt signaling pathways exist. Reinhold and colleagues reported that *Fgf-18*, an essential regulator of bone development, is a direct target of canonical Wnt signaling.(67) Moreover, these authors determined that TCF/Lef proteins bind to a consensus target sequence of the Fgf-18 promoter and, when stimulated by β-catenin, induce *Fgf-18*. However, Fgf-18 is not the only ligand of Fgf family known to be a direct target of canonical Wnt signaling. Work by Kratochwil reported that *Fgf-4* is also a target of LEF1/Wnt signaling.(68) Furthermore, a recent work by ten Berge and colleagues highlighted the interaction of Wnt and FGF signaling in coordinating growth with cell fate specification during limb development.(69) Congruent with these observations, our data suggest that an interplay between the FGF and Wnt signaling may govern the enhanced osteogenic potential and calvarial healing capacity observed in frontal bone. In perspective, it will be important to define the reciprocal relationship, interplay, and functional cooperation between these two signaling pathways so highly activated in frontal bone. Is Fgf signaling upstream of Wnt signaling or vice versa? Which of the two determines the activation of the other, and how much of the regulation is reciprocal? These are all open questions that deserve to be addressed.
