# Supplementary figures and images for: Origin Matters: Differences in Embryonic Tissue Origin and Wnt Signaling Determine the Osteogenic Potential and Healing Capacity of Frontal and Parietal Calvarial Bones
Source: J Bone Miner Res. 2009 Nov 23;25(7):1680–94. doi: 10.1359/jbmr.091116 (PMC3154006; doi:10.1359/jbmr.091116)

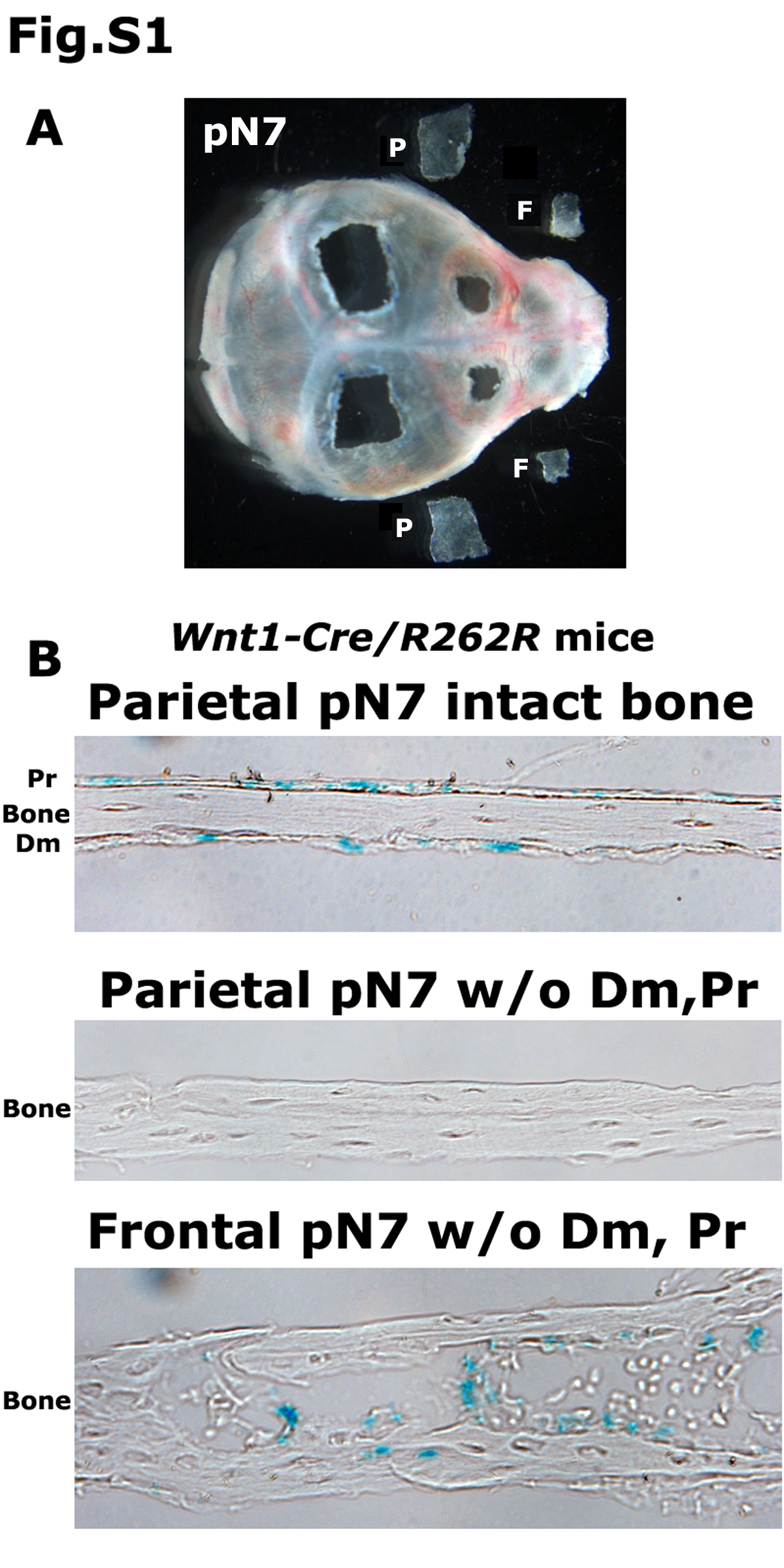

Supplement: Supplementary file 1 [file jbmr0025-1680-SD1.tif]

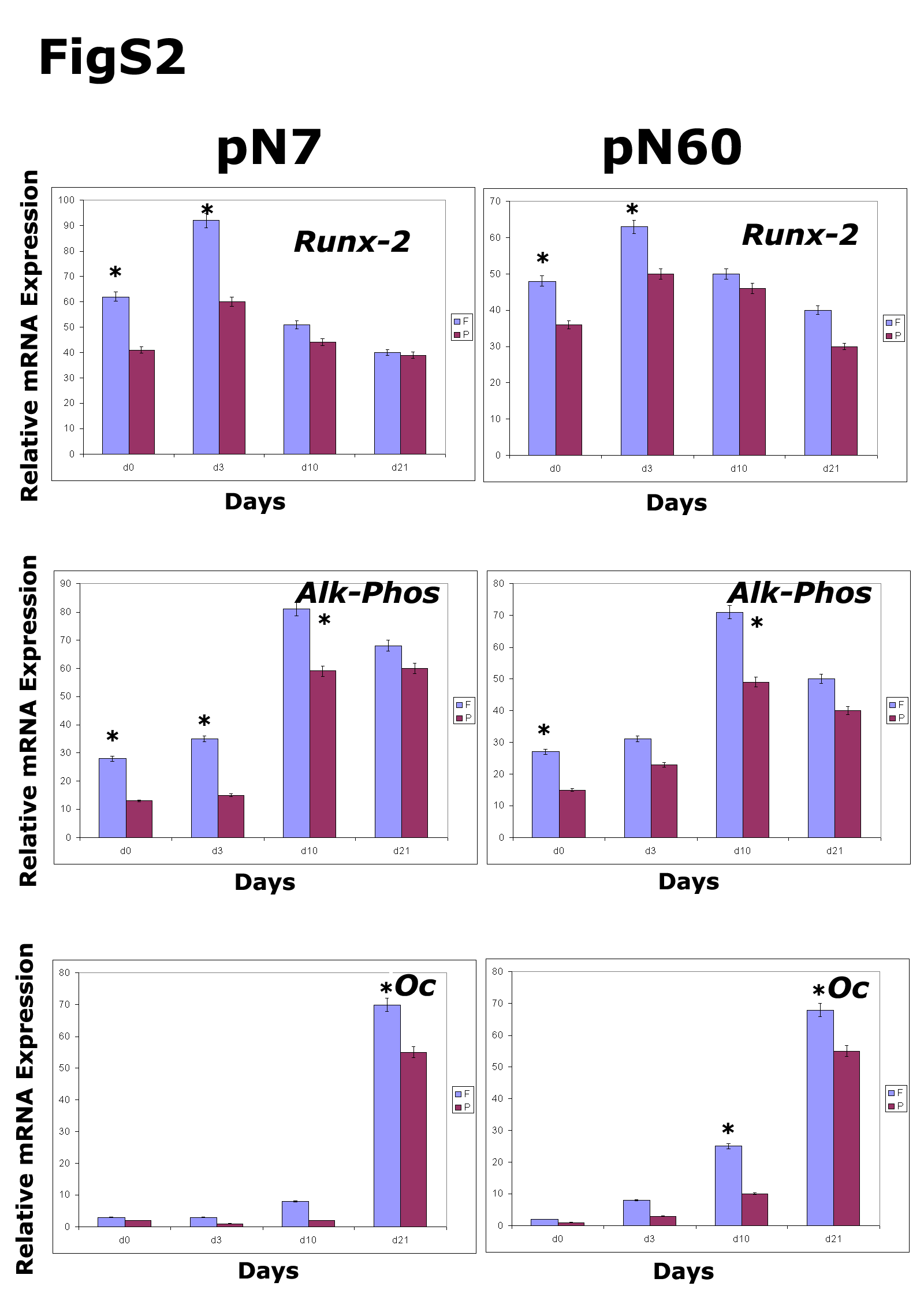

Supplement: Supplementary file 2 [file jbmr0025-1680-SD2.tif]

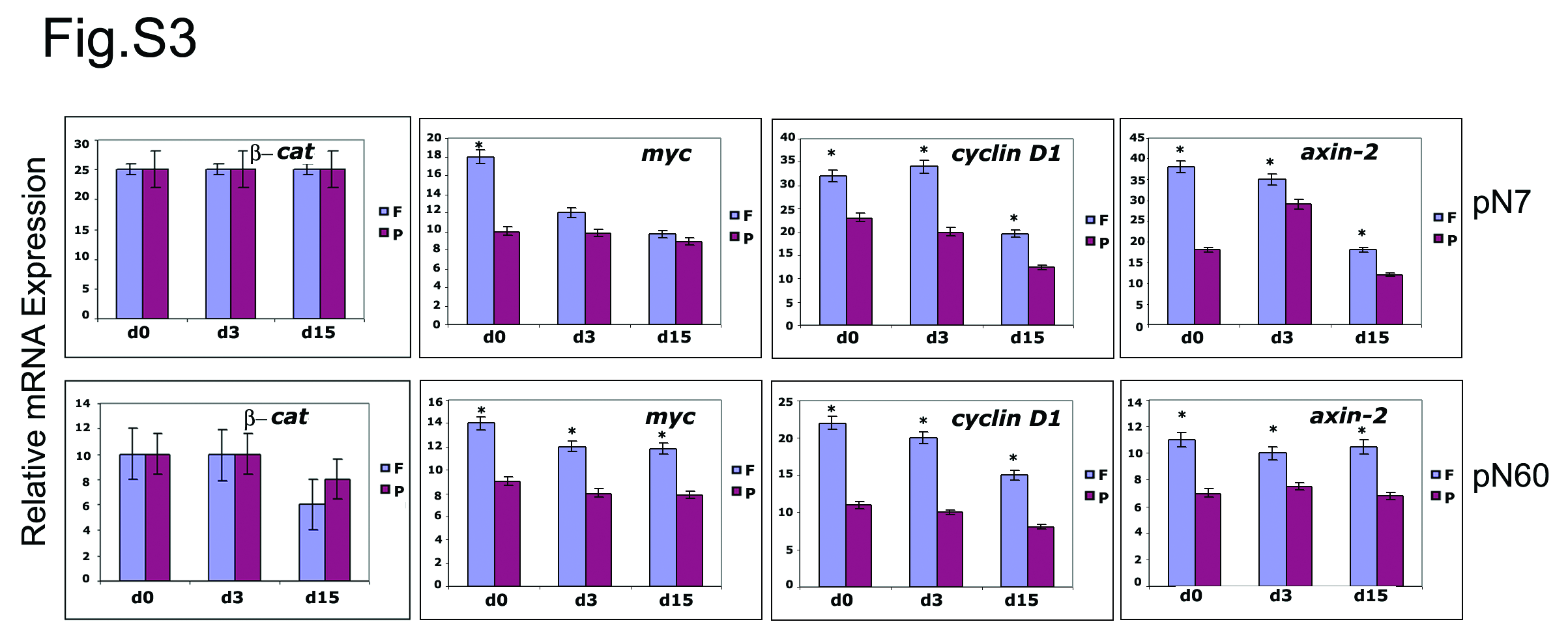

Supplement: Supplementary file 3 [file jbmr0025-1680-SD3.tif]

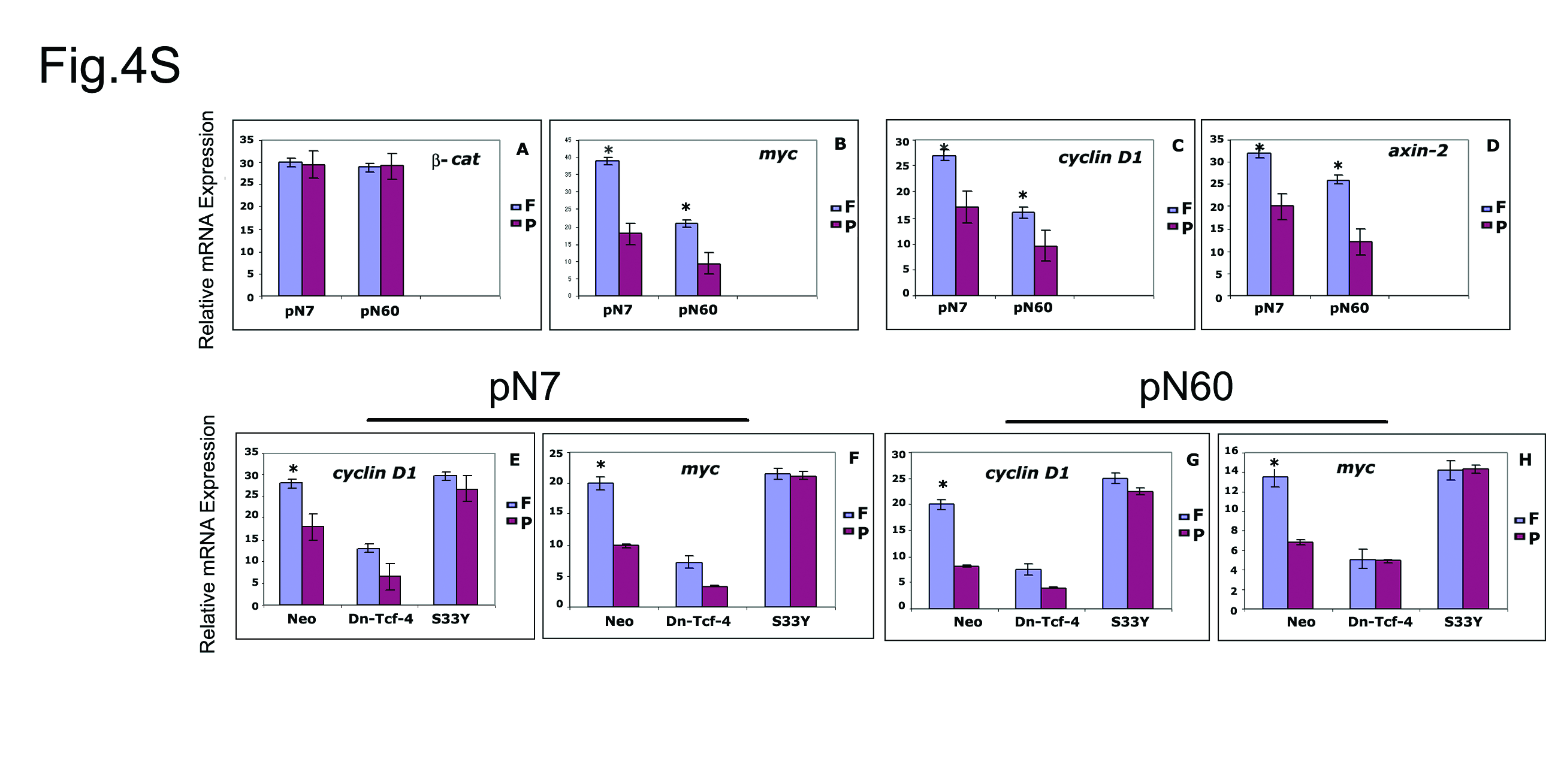

Supplement: Supplementary file 4 [file jbmr0025-1680-SD4.tif]
