# Supplementary material for: Origin Matters: Differences in Embryonic Tissue Origin and Wnt Signaling Determine the Osteogenic Potential and Healing Capacity of Frontal and Parietal Calvarial Bones
Source: J Bone Miner Res. 2009 Nov 23;25(7):1680–94. doi: 10.1359/jbmr.091116 (PMC3154006; doi:10.1359/jbmr.091116)
Supplement: Supplementary file 5 [file jbmr0025-1680-SD5.doc]

**Supplemental Figure Legend**

**Figure S1. Frontal and parietal bones osteoblasts harvesting. A,** depicts the procedure to harvest frontal and parietal bone tissues. Meticulous technique was used to exclude any suture-associated bone during the dissection of frontal and parietal bones. **B**, X-gal staining of Wnt1-Cre/R26R pN7 mice. Dura mater and pericranium stain blue when present on parietal bone (upper panel). Complete absence of lacZ positive staining is observed following meticulous removal of these neural crest-derived tissues from parietal bone (middle panel). Frontal bone depleted of pericranium and dura mater the bone tissue is uniformly lacZ positive (lower panel). Abbreviations: **F**, frontal bone; **P**, parietal bone; **Dm**, dura mater; **Pc**, pericranium; **w/o**, without

**Figure S2. Real time PCR analysis of osteogenic markers of frontal and parietal osteoblasts.** Quantitative analysis of the osteogenic markers *Runx-2,* *Alk-Phos* and *Osteocalcin* (*Oc*) performed by real-time PCR. A standard curve method of quantization was used to calculate expression of target genes relative to the house keeping gene *Gapdh*. The data correlate with results obtained by RT-PCR. The results are presented as the mean ±SD of two independent experiments. Statistical differences between the means are examined by Student’s test. A. *P value ≤0.03.

**Figure S3. Real time PCR analysis of downstream canonical Wnt target genes in frontal and parietal osteoblasts.** Quantitative analysis of endogenous levels of *β-catenin*, *cyclin D1*, *myc* and *axin-2* in pN7 and pN60 frontal and parietal osteoblasts during osteogenic differentiation. The results are presented as the mean ±SD of three independent experiments. Statistical differences between the means are examined by Student’s test. A. *P value <0.05.

**Figure S4. Real time PCR analysis of downstream canonical Wnt target genes in frontal and parietal bones. A-D,** Quantitative analysis of endogenous levels of *β-catenin*, *cyclin D1*, *myc* and *axin-2* was performed on RNA isolated from freshly harvested frontal and parietal bones (n=5). Statistical differences between the means are examined by Student’s test. A. *P value <0.03. **E-H,** real time PCR analysis of *cyclin D1*, *myc* and *axin-2* in frontal and parietal osteoblasts expressing Neo, Dn-Tcf-4, and S33Y.

**Figure S5. Differential activation of canonical Wnt signaling in frontal and parietal bones upon injury.** Two mm calvarial defects were drilled on frontal and parietal bones of pN7 and pN60 Axin-2 ^lacZ/+^reporter mice. X-gal staining of coronal sections obtained from frontal and parietal bones after 5 days injury, detected intense staining in the injured area of frontal bone of both pN7 and pN60 mice showing larger number of Wnt responsive cells. In contrast, the X-gal staining was much fainter in the injured parietal bone of pN7 and pN60 mice. Boxed areas in **A, B, G** and **H** are magnified in panels **C**, **D**, **I** and **J,** respectively. Arrows indicate the edges of calvarial defect. Panels **E, F, K** and **L** represent X-gal staining of uninjured bones. Scale bars, 200μm (**A, B, E, F, G, H, K, L**), 50μm (**C, D, I, J**).
