# Supplementary material for: Origin Matters: Differences in Embryonic Tissue Origin and Wnt Signaling Determine the Osteogenic Potential and Healing Capacity of Frontal and Parietal Calvarial Bones
Source: J Bone Miner Res. 2009 Nov 23;25(7):1680–94. doi: 10.1359/jbmr.091116 (PMC3154006; doi:10.1359/jbmr.091116)
Supplement: Supplementary file 6 [file jbmr0025-1680-SD6.doc]

**Supplemental Materials and Methods**

**Frontal and Parietal Primary Osteoblast Culture and Osteogenic Differentiation**

Frontal and parietal bones were removed under direct visualization using a dissecting microscope. The periosteum and dura mater were meticulously removed from the calvaria, and only non suture associated frontal and parietal bone was dissected free from surrounding tissue. Calvarial bones were mechanically minced and digested with 0.2% dispase II and 0.1% collagenase A (Roche Diagnostics, Indianapolis, IN) in serum-free medium. The digestion was repeated six times for 10 min each for frontal and parietal bones pN7 mice, and 15 min each for pN60 mice, at 37°C in a water bath shaker. The first two digestions were discarded. The latter four digestions were pooled, pelleted, and resuspended in growth media α-minimal essential medium (α-MEM) supplemented with 10% FCS. For the osteogenic assay first passage osteoblasts were seeded at high density in 6-well plates at 5x10^5^ per well in order to let them to reach confluency in 18/20 hours. After 18 hours, cells were incubated in osteogenic medium (day 0). At day 0 when osteogenic differentiation started, both osteoblast cultures were confluent with same cell density as assessed by cell-counting (data not shown). Osteogenic differentiation was induced by culturing cells in α-MEM supplemented with 10 % FBS, 100 IU/ml penicillin, 100 IU/ml streptomycin, 10 mM β-glycerophosphate and 100 µg/ml ascorbic acid (Sigma-Aldrich, St. Louis, MO) (Osteogenic Differentiation Medium, ODM). Osteogenic differentiation in presence of Wnt3a was performed by adding 50 ng/ml of recombinant mouse Wnt3a protein (R&D Systems, Minneapolis, MN) to the osteogenic medium. Medium was changed every three days. Only first and second passage cells were used for all experiments.

**Transfections and Infections**

Subconfluent Ψ-2 packaging cells were transfected with 10 µg of a retroviral expression vector encoding mouse Dn-Tcf-4, S33Y β-catenin (43), or with a Neo empty control vector as previously described. Stable transfected clones were isolated after two weeks of selection with 1 mg/ml G418 (Life Technologies, Gaithersburg, MA) and retroviruses were generated following expansion. Retroviruses were used to infect frontal and parietal osteoblasts for stable expression of Dn-Tcf-4 catenin S33Y or Neo control. Infections were performed as previously described (42).

**Western Blot Analysis**

For β-catenin and GSK3-β immunodetection 40 μg of cell lysate protein was resolved by 12% Tris-HCl sodium dodecyl sulfate (SDS)-polyacrylamide gel. Proteins were transferred to an Immobilon-P membrane (Millipore). Membranes were probed with mouse anti-β-catenin antibody 1:200 (sc-7963**,** Santa Cruz Biotechnology), mouse anti-active-β-catenin (anti-ABC), clone 8E7 (1:200) (Upstate.Millipore, Tamecula, CA) which specifically detect the active form of β-catenin dephosphorylated on Ser37 or Thr41 (45), rabbit anti-GSK-3β (27C10) antibody (1:1000) (Cell Signaling Technology, Danvers, MA), rabbit anti-phospho-GSK-3β (Ser9) (Cell Signaling, Danvers, MA) which detects endogenous levels of GSK-3β only when phosphorylated at Ser9. A horseradish peroxidase-conjugated secondary anti-mouse or anti-rabbit was used (Sigma Aldrich, St. Louis, MO). Immunoblotted proteins were visualized by enhanced chemiluminescence (Amersham Biosciences, Buckinghamshire, UK). Subsequently, the membranes were stripped of the antibodies and then incubated with mouse monoclonal anti-α−tubulin  antibody dilution 1:6000 (abcam.com, Cambridge, MA, USA) to control for equal loading and transfer of the samples. Where required, 50 μM LY294002 (PI3/Akt Kinase inhibitor) (Cell Signaling, Danvers, MA) was applied to the cells 1 hour before treatment with 20 ng/ml recombinant human FGF-2 (sc-4573, Santa Cruz Biotechnology, CA). Experiments were repeated three times with similar results.

**Creation and repair of calvarial defects**

Animals were anesthetized using 20 mg/kg ketamine, 1.5 mg/kg xylazine, and 0.2 mg/kg acepromazine maleate and given a preoperative dose of antibiotics (20 mg/kg cefazolin). Once adequate sedation was reached, the surgical site was cleaned with povidone-iodine. An incision was made laterally to the sagittal midline to expose the right frontal and parietal bones. Calvarial defects were created in the right frontal bone and left parietal bone of pN7 and pN60 mice (n=9/each group). The pericranium was removed using a sterile cotton swab. Using diamond coated trephine bits and saline irrigation, unilateral full-thickness calvarial defects (2 mm in diameter) was created in the non-suture associated right frontal or parietal bone. Importantly, meticulous attention was directed at avoiding injury to the dura mater.

**Evaluation of calvarial healing**

Micro-computed tomography (MicroCT) was performed on each group of mice at different time point (2, 4, and 8 weeks) prior to euthanasia for tissue processing. MicroCT images of calvaria was acquired using the GE Medical Systems eXplore RS MicroCT System. Osseous healing was quantified using the GE Microview program. A plot of bone density versus location along the diameter of the defect was obtained. Power analysis was performed by applying a two-sided Student’s t-test.

**Immunofluorescent Staining**

Cells were fixed with Methanol for 5 minutes at -20 °C then with Acetone for 2 minutes at -20 °C. Cells were washed 5 times with cold PBS then blocked with 2% normal goat serum for 30 minutes at room temperature before incubation with primary antibody mouse-anti-β-catenin (1:100, sc-7963**,** Santa Cruz Biotechnology, CA) overnight at 4 ºC followed by a fluorescein-conjugated donkey anti-mouse secondary antibody (1:800, Alexa-fluor 488, Molecular Probes, Invitrogen, Carlsbad, CA) for 1 hr at room temperature. Immunostaining with a primary normal (irrelevant) mouse IgG (sc-2025) (1:100, sc-2027, Santa Cruz Biotechnology, Inc., Santa Cruz, CA) was performed as negative control followed by incubation with anti-mouse FITC conjugated antibody. Nuclear counterstaining was performed using Vectashield H-1200 mounting medium with DAPI (Vector Laboratories, Burlingame, CA) and a Zeiss Axioplan microscope equipped with an Axiocam HRc digital camera was used for imaging.

**X-gal Staining and Pentachrome Staining**

Mouse calvaria were harvested at pN7 and pN 60 from Axin-2 ^lacZ/+^ heterozygotic mice (46) and *Wnt1-Cre/R26R* mice (2,3). Samples were fixed in 0.4% buffered phosphate-buffered paraformaldehyde overnight at 4°C. After decalcification, specimens were embedded in optimal cutting temperature (OCT), and then sectioned at 10 μm using a cryostat (Leica CM 3050S). Coronal sections were stained with X-gal for 48 hours at 37°C. For pentachrome staining to detect bony tissue, calvaria were harvested from pN7 and pN60 mice, 8 weeks after injury. Samples were fixed in 10% buffered formalin overnight at 4°C, decalcified, processed for paraffin embedding and cut in 10 μm sections. Coronal sections were histological stained using a modified Movats pentachrome staining method (47). The slides were analyzed as above.
